# Case Report: Allogeneic adipose-derived mesenchymal stem cells for severe feline chronic kidney disease

**DOI:** 10.3389/fvets.2025.1632324

**Published:** 2025-07-23

**Authors:** Yizhe Song, Yuyang Liu, Yu Yu, Ying Wang, Yunpeng Mu, Siyu Wang, Wanting Han, Hailing Zhang, Wenzheng Zhang

**Affiliations:** ^1^College of Life Sciences, Yantai University, Yantai, Shandong, China; ^2^Ainuo Companion Pet Hospital, Qingdao, Shandong, China

**Keywords:** feline, chronic kidney disease, adipose, mesenchymal stem cells, paracrine effects

## Abstract

A 10-year-old neutered male Ragdoll cat presented with symptoms of anorexia, nausea, vomiting, lethargy, and progressive weight loss. Laboratory parameters and clinical signs led to a diagnosis of International Renal Interest Society (IRIS) Chronic Kidney Disease (CKD) Stage III. Following basic supportive therapy, clinical symptoms subsided, and the patient was discharged for home management. Three months later, the cat’s condition worsened, and upon reassessment, it met the diagnostic criteria for IRIS CKD Stage IV. After 4 months of guideline-directed supportive care (in accordance with IRIS recommendations), there were no significant changes in clinical symptoms or laboratory markers, and the cat continued to exhibit a cachectic condition. Subsequently, allogeneic adipose-derived mesenchymal stem cell (AD-MSCs) therapy (2 × 10^6^ cells/kg) was initiated via intravenous (IV) infusion, administered weekly for three consecutive weeks. No adverse events, such as fever or vomiting, were observed during or after therapy. Prior to AD-MSCs therapy, the cat’s serum creatinine (SCr), blood urea nitrogen (BUN), and phosphorus (P) levels were all above the normal reference ranges. Three weeks after the final of the three AD-MSC treatments, the SCr level had returned to the reference range for CKD Stage II, while BUN and P levels showed an improving trend. The cat’s mental status and appetite also improved. Reevaluation according to IRIS CKD staging criteria confirmed functional enhancement from Stage IV to Stage II. During the subsequent follow-up period, the cat’s physical condition improved, as shown by an increase in body weight, an improved Body Condition Score (BCS), and the normalization of mucous membrane color. Concurrently, laboratory results revealed a decrease in key renal biomarkers (SCr, BUN, and P) and an amelioration of the non-regenerative anemia. This case report suggests that allogeneic AD-MSCs have potential therapeutic efficacy in felines with end-stage CKD, offering a new possibility for the treatment of terminal chronic kidney disease.

## 1 Introduction

Feline chronic kidney disease (CKD), characterized by persistent structural or functional renal abnormalities for more than 3 months in cats, is the most common metabolic condition in feline medicine (1). Epidemiological studies indicate that CKD is more prevalent in felines than in canines, with a particular susceptibility in older populations, although it can occur in cats of all ages (2). Ragdoll cats exhibit increased vulnerability to kidney diseases (3). A study of a randomly selected feline population revealed that the overall prevalence of CKD could be as high as 50%, rising to 80.9% in geriatric cats aged 15 to 20 years (4). In addition to its high prevalence, CKD poses a significant threat to feline survival. Furthermore, a study on feline mortality in England reported that kidney disease is the second most common cause of death in cats of all ages (12.1%) and the leading cause of mortality in cats aged 5 years and older (13.6%) (5). The principal histological features that drive the progression of CKD include glomerulosclerosis, tubulointerstitial inflammation, atrophy, and fibrosis (2, 6, 7). Using the International Renal Interest Society (IRIS) staging system, feline CKD is categorized into Stages I-IV based on serum creatinine concentrations, with further classification involving urine protein-to-creatinine ratio (UPC) and systolic blood pressure (SBP) (8). Histopathological findings confirm the increasing intensification of tubulointerstitial fibrosis and glomerulosclerosis in advanced stages, particularly during Stages III-IV (9). Currently, there are no widely recognized curative drugs for feline CKD. Therefore, management options are primarily limited to supportive and symptomatic care, aimed at improving the quality of life of affected cats and slowing disease progression (10, 11). Treatment options include conventional therapies and renal replacement therapies (9). However, the veterinary sector confronts two primary challenges: the lack of established pharmacological dosing protocols (12) and limited access to renal replacement therapies (11). These treatment constraints lead to insufficient clinical intervention, markedly diminishing the quality of life in cats afflicted by CKD. Recent studies emphasize mesenchymal stem cells (MSCs) as promising therapeutic agents due to their complex biological features, including anti-inflammatory, immunomodulatory, anti-apoptotic, antioxidant, anti-fibrotic, pro-angiogenic, and autophagy-regulating actions (13–15). MSCs are adult stem cells characterized by their ability to self-renew and differentiate into many lineages, commonly extracted from bone marrow, umbilical cord, adipose tissue, and various other sources (16, 17). The predominant sources employed are bone marrow-derived mesenchymal stem cells (BM-MSCs), umbilical cord-derived mesenchymal stem cells (UC-MSCs), and adipose-derived mesenchymal stem cells (AD-MSCs) (18). In comparison to other MSC types, AD-MSCs exhibit superior accessibility, increased proliferative capability, reduced immunogenicity, and a lack of ethical issues (19–21). In human clinical trials, MSC-based treatments demonstrate acceptable safety profiles and tolerability, with rising evidence of efficacy in enhancing clinical parameters (22–24). Research in veterinary medicine has concentrated on the uses of MSCs across several diseases. In felines, AD-MSCs have been studied for the treatment of chronic gingivostomatitis (25), chronic enteropathy (26, 27), asthma (28), ophthalmic disorders (29), and renal illnesses (30). Recent studies further validate the safety and feasibility of MSC treatment in felines with CKD (31–33). Nonetheless, clinical evidence assessing the safety and efficacy of allogeneic AD-MSCs for feline chronic kidney disease is still scarce. This case study involved the intravenous infusion of allogeneic AD-MSCs to a feline patient with CKD who had ongoing clinical deterioration despite adherence to IRIS guideline-recommended treatment procedures.

## 2 Case description

In August 2023, a 10-year-old neutered male Ragdoll cat presented with anorexia, nausea, vomiting, lethargy, and progressive weight loss. On physical examination, the cat weighed 6.3 kg with a body condition score (BCS) of 5/9 and had pale mucous membranes. Laboratory findings revealed a serum creatinine (SCr) of 280 μmol/L (reference interval: 71–212 μmol/L) and a blood urea nitrogen (BUN) of 16.8 mmol/L (reference interval: 5.7–12.9 mmol/L) (Supplementary Tables S1, S3). The cat was diagnosed with IRIS CKD Stage III. Following basic supportive therapy, the cat’s clinical signs subsided, and its weight stabilized at 5.7 kg. The cat was discharged for home management in mid-November.

**Figure 1:**
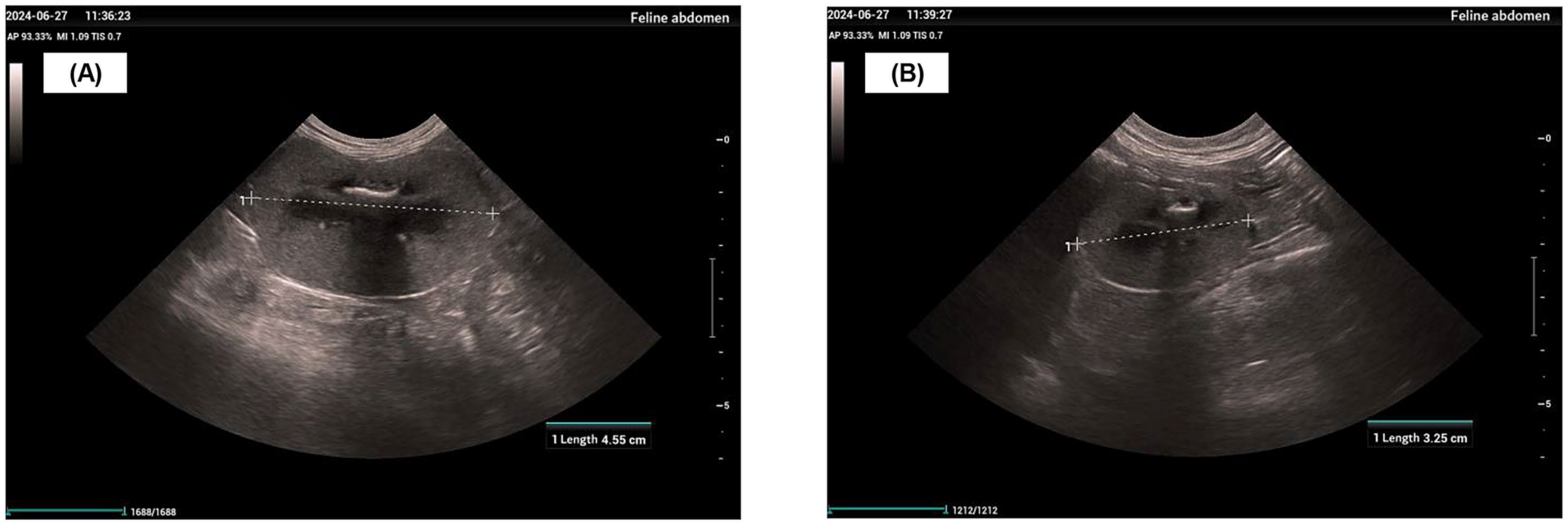
Renal ultrasound image. **(A)** Left renal length. **(B)** Right renal length.

In late June 2024, a physical examination, diagnostic imaging, and serum biochemistry were performed. The physical examination revealed a body weight of 4.6 kg, a body condition score (BCS) of 1/9, and pale mucous membranes. Diagnostic imaging identified an enlarged left kidney (4.55 cm), an atrophied right kidney (3.25 cm), thickened, hyperechoic cortices, and a loss of corticomedullary differentiation (Figures 1A,B). Serum biochemistry showed an SCr of 799 μmol/L, BUN > 46.4 mmol/L, and phosphorus (P) > 5.20 mmol/L (reference range: 1.00–2.42 mmol/L) (Supplementary Tables S1, S3). The treatment plan for the hospitalized patient consisted of lactated Ringer’s solution (200 mL SC every 24 h), maropitant (1.0 mg/kg SC every 24 h), telmisartan (1.0 mg/kg PO every 24 h), and darbepoetin alfa (1.0 μg/kg SC every 7 days), in addition to a renal prescription diet and nutraceuticals. Despite the use of IRIS guideline-recommended therapy, the cat’s SCr, BUN, and P levels remained persistently above their normal physiological reference ranges. The patient’s physical and mental status continued to be poor, with no improvement noted on physical examination or in biochemical markers. Therefore, with the owner’s consent, MSC therapy was initiated to reduce further renal damage from chronic kidney disease, alleviate clinical signs, and improve the patient’s quality of life.

**Figure 2:**
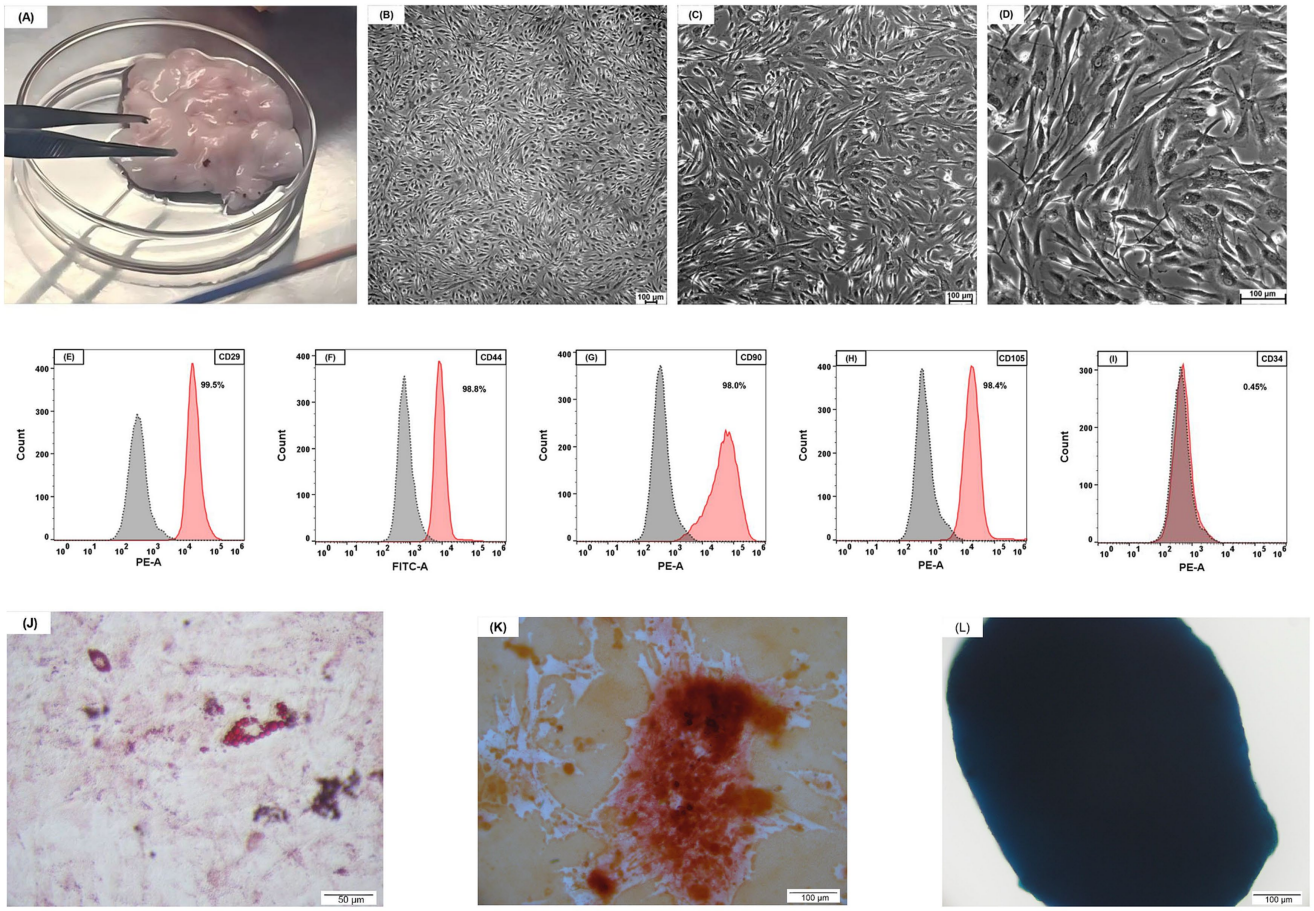
Feline adipose tissue and characterization of AD-MSCs. **(A)** Feline adipose tissue. **(B)** ×50 magnification; **(C)** ×100 magnification; **(D)** ×200 magnification; feline AD-MSCs show a spindle-shaped morphology. **(B–D)** Scale bars, 100 μm. **(E–I)** The FCM analysis has shown that feline AD-MSCs highly expressed CD29, CD44, CD90, and CD105 but did not express CD34. **(J)** Adipogenic differentiation, evaluated using Oil Red O staining. **(K)** Osteogenic differentiation, evaluated using Alizarin Red staining. **(L)** Chondrogenic differentiation, evaluated using Alcian Blue staining. **(J–L)** Scale bars: 50 μm **(J)**; 100 μm **(K,L)**.

Adipose tissue was aseptically obtained from a 9-month-old healthy Ragdoll donor cat (Figure 2A), exhibiting normal hematological parameters and negative results for infectious disease screening. The samples were rinsed three times with phosphate-buffered saline (PBS, Servicebio, China) and then deposited into centrifuge tubes containing PBS with 1% penicillin–streptomycin (P/S, Gibco, USA) before being transferred to a laminar flow hood. The adipose tissue was consecutively submerged in three centrifuge tubes containing 75% ethanol for 3 s each, then transferred to a Petri plate containing PBS for clot removal. The treated adipose tissue was subsequently transferred to new centrifuge tubes, to which PBS was added and gently inverted for mixing prior to discarding the PBS. The washing method was conducted for ten cycles. The adipose tissue was placed into new centrifuge tubes with sterile forceps and dissected into 0.5–1 mm^3^ fragments using surgical scissors. Type I collagenase (1 mg/mL; Sigma-Aldrich, USA) was then introduced, followed by incubation in a 37°C water bath with continuous agitation for 45 min to promote digestion. After digestion, an equivalent volume of complete medium was added to neutralize enzymatic activity. The complete medium was prepared using Dulbecco’s modified Eagle’s medium (DMEM; BasalMedia Technologies, China) as the base, supplemented with 10% fetal bovine serum (FBS; Excell, China), 1% penicillin–streptomycin (P/S), and basic fibroblast growth factor (bFGF; 5 ng/mL; Sino Biological, China). The cell suspension was subjected to extensive pipetting until the fibrous aggregates were fully dissociated and then filtered through a 100 μm cell strainer (pluriSelect, Germany) to remove any remaining undigested tissue pieces and cellular clumps. Following centrifugation, the cellular pellet was resuspended in the prepared DMEM complete medium and aseptically cultured in 6-well plates under controlled conditions (37°C, 5% CO₂ environment) for 7 days, with two medium exchanges conducted throughout the incubation period. Cell cultures were maintained until adherent cells reached 70–80% confluence, after which subculturing was performed. Cells were cryopreserved after five successive passages.

Microscopic analysis of MSCs revealed a typical spindle-shaped, fibroblast-like appearance (Figures 2B–D). The characteristics of the feline adipose-derived cells were investigated via flow cytometry to verify their classification as mesenchymal stem cells (MSCs). The following directly conjugated antibodies were utilized: phycoerythrin (PE) conjugated against CD29 (antibody clone TS2/16, 303004, BioLegend, USA), fluorescein isothiocyanate (FITC) conjugated against CD44 (antibody clone IM7, MA1-10229, Invitrogen, USA), PE conjugated against CD90 (antibody clone 5E10, 555596, BD Biosciences, USA), and PE conjugated against CD34 (antibody clone 1H6, 559369, BD Biosciences, USA). For CD105 phenotyping, an indirect labeling technique was utilized: cells were initially treated with an unconjugated CD105 antibody (clone SN6, 14–105782, Invitrogen, USA), followed by a PE-conjugated goat anti-mouse IgG F(ab’)₂ fragment secondary antibody (12–4010-82, Invitrogen, USA). The isotype controls comprised PE-conjugated Mouse IgG1, *κ* Isotype Control (clone MOPC-21, 555,749, BD Biosciences, USA), FITC-conjugated Rat IgG2b kappa Isotype Control (clone eB149/10H5, 11–4,031-82, Invitrogen, USA) and unconjugated Mouse IgG1 kappa Isotype Control (clone P3.6.2.8.1, 14–4,714-82, Invitrogen, USA). Cells were examined utilizing a CytoFLEX flow cytometer (Beckman Coulter, USA), and the data were assessed with FlowJo software (Tree Star, USA) (Figures 2E–I). When cultivated in certain differentiation medium, the MSCs exhibited trilineage differentiation potential into adipocytes, osteoblasts, and chondrocytes (Figures 2J–L).

**Figure 3:**
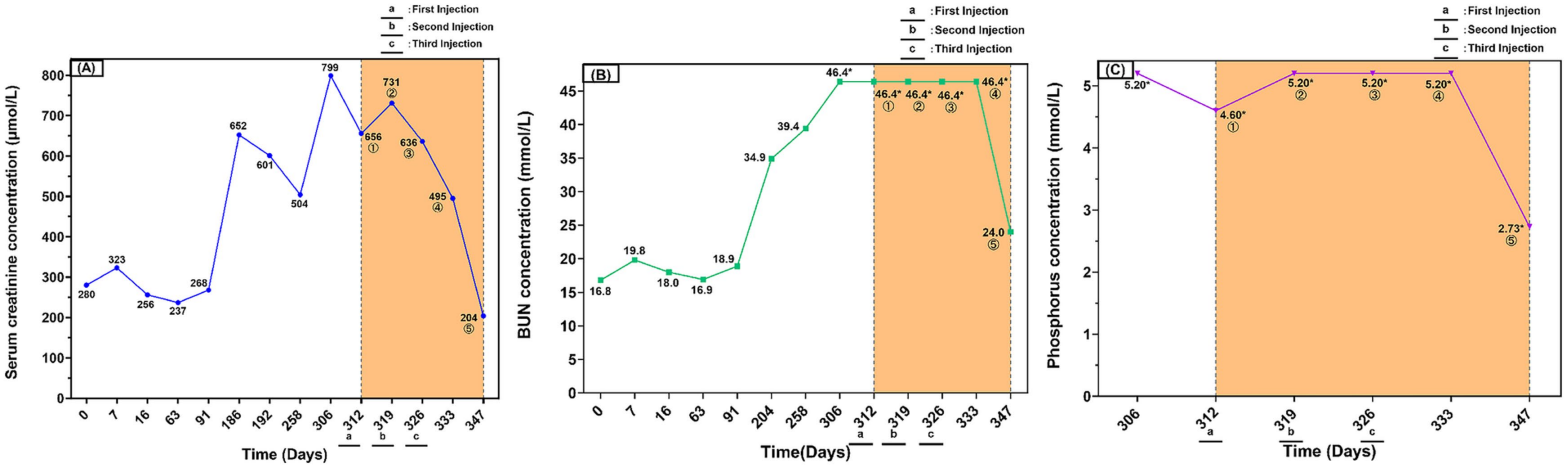
Serum creatinine, BUN, and phosphorus dynamics in a cat with chronic kidney disease during long-term conventional therapy and subsequent AD-MSCs treatment. **(A)** Serum creatinine (SCr) concentration over time (μmol/L). **(B)** Blood urea nitrogen (BUN) concentration over time (mmol/L). **(C)** Serum phosphorus (P) concentration over time (mmol/L). The yellow shaded area indicates the period of AD-MSCs treatment. The encircled numbers ①–⑤ on the figure represent measurements taken at the following time points: ① before the first injection (baseline); ② before the second injection; ③ before the third injection; ④ 1 week after the third injection; ⑤ 3 weeks after the third injection. Asterisks (*) indicate values that reached the upper limit of the instrument’s detection range. Day 0 was defined as the day treatment commenced upon the cat’s initial hospitalization in August 2023, serving as the baseline for the clinical timeline. a, b, and c indicate the time points of the first, second, and third mesenchymal stem cell infusions, respectively.

Cryopreserved AD-MSCs were thawed and cultured until the adherent cells reached 80–90% confluence. The cells were then harvested by digestion with TrypLE™ Express enzyme (1X, Red; Gibco, USA) and subsequently washed with PBS to remove culture residues. Next, the cells were resuspended in 1 mL of a Cell Refrigeration Preservation Premix (Selcell, China), which is free of dimethyl sulfoxide (DMSO) and animal-derived components. The cell suspension was immediately transferred to a sterile vial and stored protected from light at 2–8°C, with the requirement for infusion within 24 h. Prior to infusion, the cell suspension was drawn and added to an infusion bottle containing 50 mL of saline. The final dose of 8.8 × 10^6^ cells/50 mL (equivalent to 2 × 10^6^ cells/kg) was administered as a slow intravenous infusion over 60 min via an intravenous (IV) catheter. To mitigate the risk of infusion reactions, the cat was premedicated with dexamethasone (0.5 mg/kg). Vital signs were continuously monitored throughout the infusion and for 30 min post-infusion, with observation extended to 24 h. No adverse reactions, such as fever or vomiting, were observed during or after the treatment. One week after the first stem cell injection, the SCr was 731 μmol/L. One week after the second injection, it decreased to 636 μmol/L, and 1 week after the third injection, it further reduced to 495 μmol/L. Three weeks after the final injection, the SCr level dropped to 204 μmol/L, returning to the reference range for CKD Stage II (Figure 3A). The BUN and P levels, which were consistently above the normal reference range before treatment, also showed a positive trend of improvement. Three weeks after the third stem cell injection, the BUN level decreased to 24 mmol/L, and the P level fell to 2.73 mmol/L (Figures 3B,C). The patient’s mental status and appetite improved throughout the treatment period. Physical examination revealed that the cat’s body weight recovered to 5 kg, the BCS improved to 3/9, and the mucous membrane color returned to pink. Based on the laboratory data and clinical presentation, the patient was determined to have improved from CKD Stage IV to Stage II. During the subsequent follow-up period, multidimensional assessments including physical examination (PE), complete blood count (CBC), and serum biochemistry were conducted (Supplementary Tables S1–S3). The assessments revealed improvements across multiple key parameters: in physical examination, both body weight and body condition score (BCS) increased and mucous membrane color returned to normal; serum biochemistry showed a decrease in the levels of creatinine, urea nitrogen, and phosphorus; and the complete blood count reflected an amelioration of the severe renal anemia. This clinical course is consistent with a positive response to the therapy and suggests a favorable safety profile, as no significant adverse reactions were observed. Therefore, this case highlights the therapeutic potential of this regimen and provides a valuable reference for future studies on similar cases.

## 3 Discussion

Chronic kidney disease in cats is characterized by its irreversible progression and gradual onset, often evading early detection due to the absence of distinct clinical symptoms in the initial stages, resulting in diagnoses at more advanced phases (9). As a significant contributor to morbidity and mortality in older felines (5), current management strategies focus on palliative care, which includes fluid therapy (34, 35), antiemetic and orexigenic treatments (36, 37), blood pressure control, reduction of proteinuria (38), management of anemia (39), and regulation of hyperphosphatemia through dietary phosphate and protein restriction (40–43). Although these interventions may temporarily improve the quality of life, their inability to change the disease’s pathophysiology, prevent functional decline, or restore nephron structure underscores the limitations of treatment. Renal replacement therapies encompass kidney transplantation (44, 45), peritoneal dialysis (46, 47), and hemodialysis (48, 49).

Nonetheless, these methodologies present significant challenges in veterinary therapeutic practice, including ethical dilemmas, high costs, technological complexities, and the management of long-term comorbidities. Consequently, the development of innovative therapeutic strategies—particularly mechanism-targeted interventions such as stem cell-based therapies—has become a central focus in contemporary research (50–52).

MSCs show great potential in regenerative medicine for the treatment of feline diseases (53–56). For example, they have been used to treat conditions such as chronic gingivostomatitis, chronic enteropathy, asthma, ophthalmic disorders, and kidney disease. The therapeutic mechanisms of MSCs predominantly encompass two principal routes. The initial process is directed differentiation, in which a fraction of MSCs migrates to damaged renal tissues, proliferates, and differentiates into renal cells to replace the injured populations (57, 58). Nonetheless, the effectiveness of this method is limited by inadequate homing efficiency to renal locations and suboptimal post-transplant survival rates (59–61). An alternative mechanism entails MSCs providing renoprotective effects chiefly via paracrine secretion of various bioactive factors, such as vascular endothelial growth factor (VEGF), fibroblast growth factor (FGF), hepatocyte growth factor (HGF), insulin-like growth factor (IGF), transforming growth factor-beta (TGF-*β*), and interleukin-10 (IL-10). These mediators collectively facilitate renal epithelial cell proliferation, angiogenesis, anti-apoptotic mechanisms, anti-inflammatory responses, and anti-fibrotic activities (62, 63).

Adipose tissue is the predominant source of MSCs owing to their plentiful availability, little immunogenicity, and ethical acceptability, with AD-MSCs exhibiting distinct advantages in clinical applications (64, 65). AD-MSCs display a distinctive spindle-shaped fibroblast-like appearance and possess significant proliferation capability *in vitro* (66, 67). Immunophenotypic profiling verifies that feline AD-MSCs exhibit classical MSC surface markers (CD29, CD44, CD90, and CD105) and do not express the hematopoietic lineage marker CD34 (21, 68, 69), along with possessing multipotent differentiation capabilities. Although the therapeutic efficacy of AD-MSCs has been thoroughly established in rat disease models, its applicability for feline CKD has not been adequately explored in veterinary medicine.

The treatment protocol in this study was primarily based on key previous findings by Quimby et al. Their 2013 study demonstrated (70) that multiple intravenous infusions of culture-expanded AD-MSCs derived from cryopreserved adipose tissue have a good safety profile, whereas the direct use of cryopreserved cells may induce adverse reactions. Subsequently, their 2016 randomized, placebo-controlled trial (32) further established the clinical safety of a 2 × 10^6^ cells/kg dose administered via multiple intravenous infusions. Although these studies did not observe significant short-term improvements in renal function, they laid the foundation for the safe application of AD-MSCs. Therefore, this study adopted a protocol of three intravenous infusions of AD-MSCs at a dose of 2 × 10^6^ cells/kg, administered once weekly.

This case report describes clinical improvement in a cat with severe chronic kidney disease (CKD) following treatment with allogeneic mesenchymal stem cells (MSCs). No acute side effects were observed after three MSC injections. The 10-year-old male Ragdoll cat showed a decreasing trend in serum creatinine (SCr), blood urea nitrogen (BUN), and phosphorus (P) levels, along with improvements in mentation and appetite. Based on laboratory data and clinical presentation, the cat’s CKD stage improved from IRIS Stage IV to Stage II. These findings suggest that MSC therapy may represent a potential therapeutic approach for end-stage CKD.

In assessing the therapeutic results of this case, essential inquiries arise: ① Considering the restricted effectiveness of traditional medication in influencing CKD indicators, which bioactive constituents of AD-MSCs primarily facilitate their renoprotective effects? ② Current research indicates that in the treatment of feline diseases, minimal dosages of mesenchymal stem cells (1 × 10^6^ cells/kg) have restricted effectiveness, whereas elevated doses (4–5 × 10^6^ cells/kg) yield enhanced therapeutic results but may entail some side consequences (71). Establishing dynamic dose-adjustment techniques for MSCs based on disease stages to enhance the efficacy-safety balance remains a significant problem. ③ This example utilized intravenous MSC delivery, resulting in significant therapeutic benefits. Although intravenous injection is the predominant administration route, the “first-pass effect” considerably diminishes the percentage of cells that effectively engraft in damaged renal tissues. The suboptimal renal homing and engraftment efficiency is a significant obstacle to achieving optimal therapeutic efficacy. Would localized delivery methods—such as direct renal cortical (72), renal artery (73), or intraperitoneal (74) injection—improve target-site bioavailability and therapeutic efficacy?

This case report possesses numerous limitations. This single-case study lacks an adequate sample size to substantiate the therapeutic efficacy of MSC therapy. Secondly, although the paracrine mechanisms of MSCs were examined in light of existing research, this study did not explore the specific molecular pathways that underlie these mechanisms in the present context. Finally, a key limitation of this study is its open-label design, in which both the evaluating veterinarian and the cat’s owner were aware that stem cell therapy was being administered. This design carries an inherent risk of bias, particularly in the assessment of subjective outcomes such as appetite, mental status, and behavior. The observed improvements in these areas may have been influenced by the caregiver placebo effect. Therefore, future studies should incorporate blinded assessment to provide a more objective evaluation of these subjective parameters.

This case illustrates that AD-MSCs therapy was associated with improvement in essential renal function indicators (SCr, BUN, and P levels) in a feline CKD patient while also enhancing mental status and appetite, underscoring the therapeutic potential of stem cell-based interventions for end-stage CKD management. Future study should emphasize the following avenues: ① Increasing sample numbers with controlled trial designs that include long-term follow-up to clarify the enduring therapeutic efficacy and prolonged detrimental effects of AD-MSCs therapy in chronic renal disease. ② Recent studies demonstrate that preconditioning procedures augment the therapeutic efficacy of MSCs. Hypoxic preconditioning and melatonin pretreatment have demonstrated the ability to enhance the paracrine capacity of MSCs (75, 76). Future research should concentrate on examining innovative preconditioning techniques while thoroughly assessing the safety and efficacy characteristics of preconditioned MSC populations. ③ In comparison to MSCs, extracellular vesicles produced from MSCs (MSC-EVs) demonstrate reduced immunogenicity and tumorigenic potential, signifying a potentially cell-free treatment strategy. Nonetheless, their function in kidney transplantation is inadequately defined and necessitates additional research. ④ MSC therapy exhibits stage-dependent therapeutic effectiveness in CKD management, with maximum results achieved when intervention commences in the early stages of the disease. Nonetheless, their therapeutic efficacy may be limited in advanced CKD, highlighting the necessity to discover predictive biomarkers for monitoring disease progression to facilitate earlier CKD identification.

This case report illustrates that allogeneic AD-MSCs have innovative therapeutic potential for the treatment of severe CKD in feline patients. These findings warrant thorough assessment and methodical inquiry to clarify their applicability in CKD treatment strategies.

## Data Availability

The original contributions presented in the study are included in the article/Supplementary material, further inquiries can be directed to the corresponding author/s.

## Supplementary material

SUPPLEMENTARY VIDEO S1 A short video clip of a feline patient undergoing intravenous infusion of AD-MSCs.

SUPPLEMENTARY VIDEO S2 A short video clip documenting the resumption of voluntary feeding in a feline patient following AD-MSCs therapy.

SUPPLEMENTARY VIDEO S3 A short video clip demonstrating locomotor performance recovery in a feline patient following AD-MSCs therapy.

SUPPLEMENTARY VIDEO S4 A short video clip demonstrating play behavior recovery in a feline patient following AD-MSCs therapy.

SUPPLEMENTARY TABLE S1 Physical examination.

SUPPLEMENTARY TABLE S2 Complete blood count (CBC).

SUPPLEMENTARY TABLE S3 Biochemistry tests.

SUPPLEMENTARY TABLE S4 Abbreviations.

## References

[1] Bartges JW. Chronic kidney disease in dogs and cats. Vet Clin North Am Small Anim Pract. (2012) 42:669–92. doi: 10.1016/j.cvsm.2012.04.008, PMID: 22720808

[2] Brown CA Elliott J Schmiedt CW Brown SA. Chronic kidney disease in aged cats: clinical features, morphology, and proposed pathogeneses. Vet Pathol. (2016) 53:309–26. doi: 10.1177/0300985815622975, PMID: 26869151

[3] Paepe D Bavegems V Combes A Saunders JH Daminet S. Prospective evaluation of healthy ragdoll cats for chronic kidney disease by routine laboratory parameters and ultrasonography. J Feline Med Surg. (2013) 15:849–57. doi: 10.1177/1098612x13477415, PMID: 23413268 PMC11383155

[4] Marino CL Lascelles BDX Vaden SL Gruen ME Marks SL. Prevalence and classification of chronic kidney disease in cats randomly selected from four age groups and in cats recruited for degenerative joint disease studies. J Feline Med Surg. (2013) 16:465–72. doi: 10.1177/1098612x13511446 24217707 PMC4414065

[5] O'Neill DG Church DB McGreevy PD Thomson PC Brodbelt DC. Longevity and mortality of cats attending primary care veterinary practices in England. J Feline Med Surg. (2015) 17:125–33. doi: 10.1177/1098612x14536176 24925771 PMC10816413

[6] Lawson J Elliott J Wheeler-Jones C Syme H Jepson R. Renal fibrosis in feline chronic kidney disease: known mediators and mechanisms of injury. Vet J. (2015) 203:18–26. doi: 10.1016/j.tvjl.2014.10.009, PMID: 25475166

[7] Morais GB Viana DA Verdugo JM Roselló MG Porcel JO Rocha DD. Morphological characterization of CKD in cats: insights of fibrogenesis to be recognized. Microsc Res Tech. (2018) 81:46–57. doi: 10.1002/jemt.22955, PMID: 29024123

[8] Paepe D Daminet S. Feline CKD diagnosis, staging and screening - what is recommended? J Feline Med Surg. (2013) 15:15–27. doi: 10.1177/1098612x13495235, PMID: 23999183 PMC10816690

[9] McLeland SM Cianciolo RE Duncan CG Quimby JM. A comparison of biochemical and histopathologic staging in cats with chronic kidney disease. Vet Pathol. (2015) 52:524–34. doi: 10.1177/0300985814561095, PMID: 25516066

[10] Roudebush P Polzin DJ Ross SJ Towell TL Adams LG Dru FS. Therapies for feline chronic kidney disease. What is the evidence? J Feline Med Surg. (2009) 11:195–210. doi: 10.1016/j.jfms.2009.01.004, PMID: 19237135 PMC11132212

[11] Sparkes AH Caney S Chalhoub S Elliott J Finch N Gajanayake I. Isfm consensus guidelines on the diagnosis and management of feline chronic kidney disease. J Feline Med Surg. (2016) 18:219–39. doi: 10.1177/1098612x16631234, PMID: 26936494 PMC11148907

[12] De Santis F Boari A Dondi F Crisi PE. Drug-dosing adjustment in dogs and cats with chronic kidney disease. Animals-Basel. (2022) 12:262. doi: 10.3390/ani12030262, PMID: 35158584 PMC8833495

[13] Duc MH Pham P Trung QB Anh TLN Nguyen QT Trang TKP. Stem cell-based therapy for human diseases. Signal Transduct Target Ther. (2022) 7:272. doi: 10.1038/s41392-022-01134-4 35933430 PMC9357075

[14] Han YY Yang JX Fang JK Zhou YP Candi E Wang JH. The secretion profile of mesenchymal stem cells and potential applications in treating human diseases. Signal Transduct Target Ther. (2022) 7:14. doi: 10.1038/s41392-022-00932-0, PMID: 35314676 PMC8935608

[15] Wang S Lei BY Zhang E Gong PY Gu J He LL. Targeted therapy for inflammatory diseases with mesenchymal stem cells and their derived exosomes: from basic to clinics. Int J Nanomedicine. (2022) 17:1757–81. doi: 10.2147/ijn.S355366, PMID: 35469174 PMC9034888

[16] Si YL Zhao YL Hao HJ Fu XB Han WD. Mscs: biological characteristics, clinical applications and their outstanding concerns. Ageing Res Rev. (2011) 10:93–103. doi: 10.1016/j.arr.2010.08.005, PMID: 20727988

[17] Voga M Adamic N Vengust M Majdic G. Stem cells in veterinary medicine-current state and treatment options. Front Vet Sci. (2020) 7:278. doi: 10.3389/fvets.2020.00278, PMID: 32656249 PMC7326035

[18] Mazini L Rochette L Amine M Malka G. Regenerative capacity of adipose derived stem cells (Adscs), comparison with mesenchymal stem cells (Mscs). Int J Mol Sci. (2019) 20:2523. doi: 10.3390/ijms20102523, PMID: 31121953 PMC6566837

[19] Qin Y Ge GR Yang P Wang LL Qiao YS Pan GQ. An update on adipose-derived stem cells for regenerative medicine: where challenge meets opportunity. Adv Sci. (2023) 10:2207334. doi: 10.1002/advs.202207334, PMID: 37162248 PMC10369252

[20] Bunnell B. Adipose tissue-derived mesenchymal stem cells. Cells. (2021) 10:3433. doi: 10.3390/cells10123433, PMID: 34943941 PMC8700397

[21] MarxCamila SilveiraMaiele D Beyer N. Adipose-derived stem cells in veterinary medicine: characterization and therapeutic applications. Stem Cells Dev. (2015) 24:803–13. doi: 10.1089/scd.2014.0407 25556829

[22] Packham DK Fraser IR Kerr PG Segal KR. Allogeneic mesenchymal precursor cells (Mpc) in diabetic nephropathy: a randomized, placebo-controlled, dose escalation study. EBioMedicine. (2016) 12:263–9. doi: 10.1016/j.ebiom.2016.09.011, PMID: 27743903 PMC5078602

[23] Sávio-Silva C Beyerstedt S Soinski-Sousa PE Casaro EB Balby-Rocha MTA Simplício A. Mesenchymal stem cell therapy for diabetic kidney disease: a review of the studies using syngeneic, autologous, allogeneic, and xenogeneic cells. Stem Cells Int. (2020) 2020:8833725. doi: 10.1155/2020/8833725 33505469 PMC7812547

[24] Habiba UE Khan N Greene DL Shamim S Umer A. The therapeutic effect of mesenchymal stem cells in diabetic kidney disease. J Mol Med Berl. (2024) 102:537–70. doi: 10.1007/s00109-024-02432-w, PMID: 38418620 PMC10963471

[25] Arzi B Mills-Ko E Verstraete FJM Kol A Walker NJ Badgley MR. Therapeutic efficacy of fresh, autologous mesenchymal stem cells for severe refractory Gingivostomatitis in cats. Stem Cells Transl Med. (2016) 5:75–86. doi: 10.5966/sctm.2015-0127, PMID: 26582907 PMC4704876

[26] Xie QY Gong SS Cao JT Li AY Kulyar MF Wang BY. Mesenchymal stem cells: a novel therapeutic approach for feline inflammatory bowel disease. Stem Cell Res Ther. (2024) 15:409. doi: 10.1186/s13287-024-04038-y, PMID: 39522034 PMC11550560

[27] Webb TL Webb CB. Comparing adipose-derived mesenchymal stem cells with prednisolone for the treatment of feline inflammatory bowel disease. J Feline Med Surg. (2022) 24:E244–50. doi: 10.1177/1098612x221104053 35713592 PMC10812281

[28] Trzil JE Masseau I Webb TL Chang CH Dodam JR Liu H. Intravenous adipose-derived mesenchymal stem cell therapy for the treatment of feline asthma: a pilot study. J Feline Med Surg. (2016) 18:981–90. doi: 10.1177/1098612x15604351, PMID: 26384398 PMC11112236

[29] Villatoro AJ Claros S Fernández V Alcoholado C Fariñas F Moreno A. Safety and efficacy of the mesenchymal stem cell in feline eosinophilic keratitis treatment. BMC Vet Res. (2018) 14:116. doi: 10.1186/s12917-018-1413-4, PMID: 29587744 PMC5870249

[30] Rosselli DD Mumaw JL Dickerson V Brown CA Brown SA Schmiedt CW. Efficacy of allogeneic mesenchymal stem cell Administration in a Model of acute ischemic kidney injury in cats. Res Vet Sci. (2016) 108:18–24. doi: 10.1016/j.rvsc.2016.07.003, PMID: 27663365

[31] Vidane AS Pinheiro AO Casals JB Passarelli D Hage M Bueno RS. Transplantation of amniotic membrane-derived multipotent cells ameliorates and delays the progression of chronic kidney disease in cats. Reprod Domest Anim. (2017) 52:316–26. doi: 10.1111/rda.12846, PMID: 27774657

[32] Quimby JM Webb TL Randall E Marolf A Valdes-Martinez A Dow SW. Assessment of intravenous adipose-derived allogeneic mesenchymal stem cells for the treatment of feline chronic kidney disease: a randomized, placebo-controlled clinical trial in eight cats. J Feline Med Surg. (2016) 18:165–71. doi: 10.1177/1098612x15576980, PMID: 25784460 PMC11149004

[33] Zacharias S Welty MB Sand TT Black LL. Impact of allogeneic feline uterine-derived mesenchymal stromal cell intravenous treatment on renal function of nephrectomized cats with chronic kidney disease. Res Vet Sci. (2021) 141:33–41. doi: 10.1016/j.rvsc.2021.09.015, PMID: 34653723

[34] Langston C Gordon D. Effects of IV fluids in dogs and cats with kidney failure. Front Vet Sci. (2021) 8:659960. doi: 10.3389/fvets.2021.659960, PMID: 33959654 PMC8093391

[35] Cooley CM Quimby JM Caney SMA Sieberg LG. Survey of owner subcutaneous fluid practices in cats with chronic kidney disease. J Feline Med Surg. (2018) 20:884–90. doi: 10.1177/1098612x17732677, PMID: 28948902 PMC11129241

[36] Quimby JM Lunn KF. Mirtazapine as an appetite stimulant and anti-emetic in cats with chronic kidney disease: a masked placebo-controlled crossover clinical trial. Vet J. (2013) 197:651–5. doi: 10.1016/j.tvjl.2013.05.048, PMID: 23838205

[37] Quimby JM Brock WT Moses K Bolotin D Patricelli K. Chronic use of Maropitant for the Management of Vomiting and Inappetence in cats with chronic kidney disease: a blinded, placebo-controlled clinical trial. J Feline Med Surg. (2015) 17:692–7. doi: 10.1177/1098612x14555441, PMID: 25336450 PMC11104052

[38] Sent U Gössl R Elliott J Syme H Zimmering T. Comparison of efficacy of long-term oral treatment with telmisartan and benazepril in cats with chronic kidney disease. J Vet Intern Med. (2015) 29:1479–87. doi: 10.1111/jvim.13639, PMID: 26474314 PMC4895689

[39] Chalhoub S Langston CE Farrelly J. The use of Darbepoetin to stimulate erythropoiesis in Anemia of chronic kidney disease in cats: 25 cases. J Vet Intern Med. (2012) 26:363–9. doi: 10.1111/j.1939-1676.2011.00864.x, PMID: 22296687

[40] Polzin DJ Churchill JA. Controversies in veterinary nephrology: renal diets are indicated for cats with international renal interest society chronic kidney disease stages 2 to 4: the pro view. Vet Clin North Am Small Anim Pract. (2016) 46:1049–65. doi: 10.1016/j.cvsm.2016.06.005, PMID: 27485277

[41] Dobenecker B Webel A Reese S Kienzle E. Effect of a high phosphorus diet on indicators of renal health in cats. J Feline Med Surg. (2018) 20:339–43. doi: 10.1177/1098612x17710589, PMID: 28569079 PMC11129231

[42] Schauf S Coltherd JC Atwal J Gilham M Carvell-Miller LJ Renfrew H. Clinical progression of cats with early-stage chronic kidney disease fed diets with varying protein and phosphorus contents and calcium to phosphorus ratios. J Vet Intern Med. (2021) 35:2797–811. doi: 10.1111/jvim.16263, PMID: 34545958 PMC8692190

[43] Stockman J. Dietary phosphorus and renal disease in cats: where are we? J Feline Med Surg. (2024) 26:10986. doi: 10.1177/1098612x241283355, PMID: 39376053 PMC11529143

[44] Aronson LR. Update on the current status of kidney transplantation for chronic kidney disease in animals. Vet Clin North Am Small Anim Pract. (2016) 46:1193–218. doi: 10.1016/j.cvsm.2016.06.013, PMID: 27593577

[45] Yeates JW. Ethical considerations in feline renal transplantation. Vet J. (2014) 202:405–7. doi: 10.1016/j.tvjl.2014.10.006, PMID: 25453241

[46] Cooper RL Labato MA. Peritoneal dialysis in veterinary medicine. Vet Clin North Am Small Anim Pract. (2011) 41:91–113. doi: 10.1016/j.cvsm.2010.10.002, PMID: 21251512

[47] Nikitidou O Peppa VI Leivaditis K Eleftheriadis T Zarogiannis SG Liakopoulos V. Animal models in peritoneal dialysis. Front Physiol. (2015) 6:244. doi: 10.3389/fphys.2015.00244 26388781 PMC4555018

[48] Segev G Foster JD Francey T Langston C Schweighauser A Cowgill LD. International renal interest society best practice consensus guidelines for intermittent hemodialysis in dogs and cats. Vet J. (2024) 305:106092. doi: 10.1016/j.tvjl.2024.106092, PMID: 38442779

[49] Bloom CA Labato MA. Intermittent hemodialysis for small animals. Vet Clin North Am Small Anim Pract. (2011) 41:115–33. doi: 10.1016/j.cvsm.2010.11.001, PMID: 21251513

[50] Pittenger MF Discher DE Péault BM Phinney DG Hare JM Caplan AI. Mesenchymal stem cell perspective: cell biology to clinical progress. NPJ Regen Med. (2019) 4:22. doi: 10.1038/s41536-019-0083-6, PMID: 31815001 PMC6889290

[51] Yu H L X-z Z Y H Y Chang F D J. Mesenchymal stem cells for regenerative medicine. Cells. (2019) 8:886. doi: 10.3390/cells8080886 31412678 PMC6721852

[52] Hoffman AM Dow SW. Concise review: stem cell trials using companion animal disease models. Stem Cells. (2016) 34:1709–29. doi: 10.1002/stem.2377, PMID: 27066769

[53] El-Husseiny HM Mady EA Helal MAY Tanaka R. The pivotal role of stem cells in veterinary regenerative medicine and tissue engineering. Vet Sci. (2022) 9:648. doi: 10.3390/vetsci9110648, PMID: 36423096 PMC9698002

[54] Baouche M Ochota M Locatelli Y Mermillod P Nizanski W. Mesenchymal stem cells: generalities and clinical significance in feline and canine medicine. Animals Basel. (2023) 13:1903. doi: 10.3390/ani13121903 37370414 PMC10295255

[55] Webb TL Webb CB. Scoping review of the use of mesenchymal stem and stromal cell products in cats, part 2: current scope and efficacy. J Am Vet Med Assoc. (2024) 262:S24-30. doi: 10.2460/javma.24.02.0080 38565137

[56] Jw Z Pezzanite L Chow L Meagan R Steven D. Evaluation of stem cell therapies in companion animal disease models: a concise review (2015-2023). Stem Cells. (2024) 42:677–705. doi: 10.1093/stmcls/sxae034 38795363 PMC13032169

[57] Karp J Sock Leng T G. Mesenchymal stem cell homing: the devil is in the details. Cell Stem Cell. (2009) 4:206–16. doi: 10.1016/j.stem.2009.02.001 19265660

[58] Nitzsche F Muller C Lukomska B Jolkkonen J Deten A Boltze J. Concise review: Msc adhesion Cascade-insights into homing and Transendothelial migration. Stem Cells. (2017) 35:1446–60. doi: 10.1002/stem.2614, PMID: 28316123

[59] Eirin A Lerman LO. Mesenchymal stem cell treatment for chronic renal failure. Stem Cell Res Ther. (2014) 5:83. doi: 10.1186/scrt472, PMID: 25158205 PMC4097822

[60] Liu DW Cheng F Pan SK Liu ZS. Stem cells: a potential treatment option for kidney diseases. Stem Cell Res Ther. (2020) 11. doi: 10.1186/s13287-020-01751-2, PMID: 32586408 PMC7318741

[61] Marcheque J Bussolati B Csete M Perin L. Concise reviews: stem cells and kidney regeneration: an update. Stem Cells Transl Med. (2019) 8:82–92. doi: 10.1002/sctm.18-0115, PMID: 30302937 PMC6312445

[62] Li JP Wu MT He LJ. Immunomodulatory effects of mesenchymal stem cell therapy in chronic kidney disease: a literature review. BMC Nephrol. (2025) 26:107. doi: 10.1186/s12882-025-04029-y, PMID: 40033224 PMC11874639

[63] Chen FK Chen NN Xia CJ Wang HY Shao LS Zhou C. Mesenchymal stem cell therapy in kidney diseases: potential and challenges. Cell Transplant. (2023) 32:09636897231164251. doi: 10.1177/09636897231164251, PMID: 37013255 PMC10074620

[64] Zhao LN Johnson T Liu D. Therapeutic angiogenesis of adipose-derived stem cells for ischemic diseases. Stem Cell Res Ther. (2017) 8:125. doi: 10.1186/s13287-017-0578-2, PMID: 28583178 PMC5460534

[65] Bukowska J Szóstek-Mioduchowska A Marta K Katarzyna W Sylwia M Barbara G-K. Adipose-derived stromal/stem cells from large animal models: from basic to applied science. Stem Cell Rev Rep. (2020) 17:719–38. doi: 10.1007/s12015-020-10049-y 33025392 PMC8166671

[66] Algorta A Rody A Analía R Benavides U Maisonnave J Yaneselli K. Morphologic, proliferative, and cytogenetic changes during in vitro propagation of cat adipose tissue-derived mesenchymal stromal/stem cells. Animals Basel. (2024) 14:2408. doi: 10.3390/ani14162408 39199942 PMC11350862

[67] Susanne K Eichler H Stoeve J Klüter H Bieback K. Comparative analysis of mesenchymal stem cells from bone marrow, umbilical cord blood, or adipose tissue. Stem Cells. (2006) 24:1294–301. doi: 10.1634/stemcells.2005-0342 16410387

[68] Metka V Valerija K Majdič G. Comparison of canine and feline adipose-derived mesenchymal stem cells/medicinal signaling cells with regard to cell surface marker expression, viability, proliferation, and differentiation potential. Front Vet Sci. (2021) 7:610240. doi: 10.3389/fvets.2020.610240 33521084 PMC7838367

[69] Kim HR Lee J Byeon JS Gu NY Lee J Cho IS. Extensive characterization of feline intra-abdominal adipose-derived mesenchymal stem cells. J Vet Sci. (2017) 18:299–306. doi: 10.4142/jvs.2017.18.3.299, PMID: 27456770 PMC5639082

[70] Quimby JM Webb TL Habenicht LM Dow SW. Safety and efficacy of intravenous infusion of allogeneic cryopreserved mesenchymal stem cells for treatment of chronic kidney disease in cats: results of three sequential pilot studies. Stem Cell Res Ther. (2013) 4:48. doi: 10.1186/scrt198, PMID: 23632128 PMC3707049

[71] dos Santos LG Ferreira PI Krause A. Mesenchymal stem cell transplantation: systematic review, Meta-analysis and clinical applications for acute kidney injury and chronic kidney disease in dogs and cats. Res Vet Sci. (2024) 175:105313. doi: 10.1016/j.rvsc.2024.105313, PMID: 38851051

[72] Quimby J Webb T Gibbons D Dow S. Evaluation of intrarenal mesenchymal stem cell injection for treatment of chronic kidney disease in cats: a pilot study. J Feline Med Surg. (2011) 13:418–26. doi: 10.1016/j.jfms.2011.01.005, PMID: 21334237 PMC10832716

[73] Abigail LT Berent A Weisse C Langston C. Intra-arterial renal infusion of autologous mesenchymal stem cells for treatment of chronic kidney disease in cats: phase I clinical trial. J Vet Intern Med. (2019) 33:1353–61. doi: 10.1111/jvim.15486 30924554 PMC6524114

[74] Maciej P Nathan CN Koehl K Miller R Kaneene J John MK. Safety of intraperitoneal injection of adipose tissue-derived autologous mesenchymal stem cells in cats. J Vet Intern Med. (2015) 30:157–63. doi: 10.1111/jvim.13655 26512713 PMC4913639

[75] Naoki I Nakashima A Doi S Ken Y Satoshi M Ryoma K. Hypoxia-preconditioned mesenchymal stem cells prevent renal fibrosis and inflammation in ischemia-reperfusion rats. Stem Cell Res Ther. (2020) 11:130. doi: 10.1186/s13287-020-01642-6 32197638 PMC7083035

[76] Lingfei Z Chenxia H Ping Z Hua J Jianghua C. Melatonin preconditioning is an effective strategy for mesenchymal stem cell-based therapy for kidney disease. J Cell Mol Med. (2019) 24:25–33. doi: 10.1111/jcmm.14769 31747719 PMC6933322

