# Supplementary material for: Case Report: Allogeneic adipose-derived mesenchymal stem cells for severe feline chronic kidney disease
Source: Front Vet Sci. 2025 Jul 23;12:1632324. doi: 10.3389/fvets.2025.1632324 (PMC12327094; doi:10.3389/fvets.2025.1632324)
Supplement: SUPPLEMENTARY TABLE S1 — Physical examination. [file Data_Sheet_1.pdf]

Supplementary Table 1. Physical Examination

Time (DAY)	BODY WEIGHT (KG)	HEART RATE	RESPIRATORY RATE	BODY TEMP (°C)	MUCOUS MEMBRANE COLOR	BCS (9-POINT SCALE)	CRT
0	6.3	144	24	38.7	Pale	5	<2
7	5.8	124	26	38.8	Pale	3	<2
16	5.6	128	25	38.8	Pale	3	<2
63	5.6	124	31	38.8	Pale	3	<2
83	5.7	128	24	38.8	Pale	3	<2
186	5.0	160	31	38.7	Pale	3	<2
192	5.0	168	30	38.9	Pale	3	<2
258	5.2	142	28	38.9	Pale	3	<2
306	4.6	129	26	38.8	Pale	1	<2
312	4.7	142	26	38.6	Pale	1	<2
319	4.8	160	24	38.5	Pale	1	<2
326	4.7	162	26	38.7	Pale	1	<2
333	5.0	128	26	38.9	Pale	1	<2
347	5.0	124	24	38.4	Pink	3	<2
361	5.1	138	26	38.8	Pink	3	<2
375	5.0	134	24	38.9	Pink	3	<2
381	5.1	128	26	38.6	Pink	3	<2
386	5.0	124	28	38.7	Pink	3	<2
396	5.2	138	26	38.8	Pink	3	<2

Supplementary Table 1: The patient's physical examination information from hospital admission to the end of the treatment and the follow-up period.In the temporal sequence, the time points of 312, 319, and 326 days correspond to the injection times of AD-MSCs. The follow-up period commences after 347 days.

Supplementary Table 2. Complete Blood Count (CBC)

TIME (DAY)	63	347	361	375
WBC ($\times 10^3/\mu L$)	20.8	20.9	12.4	21.1
RBC ($\times 10^6/\mu L$	5.31	5.00	4.51	7.28
HGB (g/dL)	7.5	4.9	5.3	9.2
HCT (%)	23.9	16.7	17.1	30.1
MCV (fL)	45.0	33.4	37.9	41.3
MCH (pg)	14.1	9.8	11.8	12.6
MCHC (g/dL)	31.4	29.3	31.0	30.6
PLT ($\times 10^3/\mu L$)	293	693	429	505
LYM (%)	30.3	13.4	18.9	6.8
OTHR (%)	66.5	83.8	80.0	91.7
EO (%)	3.2	2.8	1.1	1.5
LYM# ($\times 10^3/\mu L$)	6.3	2.8	2.3	1.4
OTHR# ($\times 10^3/\mu L$)	13.8	17.5	9.9	19.3
EO# ($\times 10^3/\mu L$)	0.7	0.6	0.2	0.4

Supplementary Table 2: The complete blood cell count results from the time of the patient's hospital admission to the end of the treatment and throughout the follow-up period.

Supplementary Table 3. Biochemistry Tests

Time (DAY)	Serum Creatinine (SCr)	Blood Urea Nitrogen (BUN)	Phosphate (P)
0	280	16.8	/
7	323	19.8	/
16	256	18.0	/
63	237	16.9	/
91	268	18.9	/
186	652	/	/
192	601	/	/
258	504	39.4	/
306	799	>46.4	>5.20
312	656	>46.4	4.6
319	731	>46.4	>5.20
326	636	>46.4	>5.20
333	495	>46.4	>5.20
347	204	24.0	2.73
381	256	18.0	/

Supplementary Table 3: The biochemistry tests results from the time of the patient's

hospital admission to the end of the treatment and throughout the follow-up period.

Supplementary Table 4. Abbreviations

CKD	Chronic Kidney Disease
IRIS	International Renal Interest Society
MSCs	Mesenchymal Stem Cells
AD-MSCs	Adipose-derived Mesenchymal Stem Cells
BM-MSC	Bone Marrow Mesenchymal Stem Cell
UC-MSCs	Umbilical Cord Mesenchymal Stem Cells
SCr	Serum Creatinine
BUN	Blood Urea Nitrogen
P	Phosphorus
UPC	Urine Protein-to-Creatinine Ratio
SBP	Systolic Blood Pressure
PE	Physical Examination
BCS	Body Condition Score
CBC	Complete Blood Count
RBC	Red Blood Cell
HGB	Hemoglobin
HCT	Hematocrit
PBS	Phosphate-Buffered Saline
P/S	Penicillin-Streptomycin
DMEM	Dulbecco's Modified Eagle's Medium
FBS	Fetal Bovine Serum
bFGF	Basic Fibroblast Growth Factor
VEGF	Vascular Endothelial Growth Factor
FGF	Fibroblast Growth Factor
HGF	Hepatocyte Growth Factor
IGF	Insulin-like Growth Factor
TGF-β	Transforming Growth Factor-β
IL-10	Interleukin-10
MSC-Evs	Mesenchymal Stem Cell-derived Extracellular Vesicles
IV	Intravenous

Supplementary Table 4: The abbreviations employed in this paper and their corresponding full terms.
